# Brown Spiders’ Phospholipases-D with Potential Therapeutic Applications: Functional Assessment of Mutant Isoforms

**DOI:** 10.3390/biomedicines9030320

**Published:** 2021-03-21

**Authors:** Thaís Pereira da Silva, Fernando Jacomini de Castro, Larissa Vuitika, Nayanne Louise Costacurta Polli, Bruno César Antunes, Marianna Bóia-Ferreira, João Carlos Minozzo, Ricardo Barros Mariutti, Fernando Hitomi Matsubara, Raghuvir Krishnaswamy Arni, Ana Carolina Martins Wille, Andrea Senff-Ribeiro, Luiza Helena Gremski, Silvio Sanches Veiga

**Affiliations:** 1 Departamento de Biologia Celular, Universidade Federal do Paraná, Curitiba 81530-900, Paraná, Brazil; thaiscwb@yahoo.com.br (T.P.d.S.); ferjacomini.fj@gmail.com (F.J.d.C.); larissavuitika@hotmail.com (L.V.); nayannepolli@gmail.com (N.L.C.P.); brunocesarantunes@hotmail.com (B.C.A.); marianna.boia@gmail.com (M.B.-F.); fernando_matsubara@hotmail.com (F.H.M.); senffribeiro@gmail.com (A.S.-R.); luizagremski@ufpr.br (L.H.G.); 2 Centro de Produção e Pesquisa de Imunobiológicos (CPPI), Piraquara 83302-200, Paraná, Brazil; jminozzo@yahoo.com.br; 3 Departamento de Física, Centro Multiusuário de Inovação Biomolecular, Universidade Estadual Paulista (UNESP), São José do Rio Preto 15054-000, São Paulo, Brazil; ricardomariutti@yahoo.com.br (R.B.M.); raghuvir.arni@unesp.br (R.K.A.); 4 Departamento de Biologia Estrutural, Molecular e Genética, Universidade Estadual de Ponta Grossa, Ponta Grossa 84030-900, Paraná, Brazil; anacarolina.wille@yahoo.com.br

**Keywords:** *Loxosceles*, venom enzymes, phospholipases-D, mutant phospholipases-D, site-directed mutations

## Abstract

Phospholipases-D (PLDs) found in *Loxosceles* spiders’ venoms are responsible for the dermonecrosis triggered by envenomation. PLDs can also induce other local and systemic effects, such as massive inflammatory response, edema, and hemolysis. Recombinant PLDs reproduce all of the deleterious effects induced by *Loxosceles* whole venoms. Herein, wild type and mutant PLDs of two species involved in accidents—*L. gaucho* and *L. laeta*—were recombinantly expressed and characterized. The mutations are related to amino acid residues relevant for catalysis (H12-H47), magnesium ion coordination (E32-D34) and binding to phospholipid substrates (Y228 and Y228-Y229-W230). Circular dichroism and structural data demonstrated that the mutant isoforms did not undergo significant structural changes. Immunoassays showed that mutant PLDs exhibit conserved epitopes and kept their antigenic properties despite the mutations. Both in vitro (sphingomyelinase activity and hemolysis) and in vivo (capillary permeability, dermonecrotic activity, and histopathological analysis) assays showed that the PLDs with mutations H12-H47, E32-D34, and Y228-Y229-W230 displayed only residual activities. Results indicate that these mutant toxins are suitable for use as antigens to obtain neutralizing antisera with enhanced properties since they will be based on the most deleterious toxins in the venom and without causing severe harmful effects to the animals in which these sera are produced.

## 1. Introduction

Spiders of the *Loxosceles* genus are commonly referred to as brown spiders and have been broadly implicated in accidents with humans [1,2]. In Brazil, *Loxosceles* spiders are considered a public health issue, being responsible for approximately 8000 accidents each year over the period of 2017–2019 [3]. *Loxosceles intermedia*, *Loxosceles gaucho* and *Loxosceles laeta* are the most important species from this medical standpoint. Envenomation caused by *Loxosceles* spiders generates loxoscelism is the term used to describe the clinical findings observed following the bites, and is categorized into two variants: cutaneous and systemic. Cutaneous loxoscelism is represented by local reactions seen near or at the bite site, such as swelling, erythema, ecchymosis, cutaneous rash, inflammatory response, and dermonecrotic lesion with gravitational spreading. Systemic loxoscelism can result in thrombocytopenia, intravascular hemolysis, acute renal failure, and in some cases, can lead to death in patients [1,2,4].

*Loxosceles* spiders’ venoms have a colorless appearance and consist of a complex mixture enriched in proteins and glycoproteins with molecular masses ranging from 3–45 kDa [1,2,4]. Amongst the great diversity of toxins found in these venoms, the Phospholipases-D (PLDs) are undoubtedly the most well-studied and functionally well-characterized molecules. PLDs cause the majority of the pathological effects triggered by *Loxosceles* crude venoms such as massive inflammatory response and hemolysis, in addition to dermonecrosis, which is the reason why they are also known as dermonecrotic toxins. PLDs present in the venom of *Loxosceles* spiders were initially characterized as sphingomyelinases-D based on their ability to cleave sphingomyelin, resulting in choline and ceramide-1-phosphate [5,6]. Currently, these molecules are classified as a phospholipases-D due to the wide spectrum of lipids that they can cleave such as lysophosphatidylcholine, lysophosphatidylethanolamine, lysophosphatidylserine, lysophosphatidylinositol, lyso- platelet activating factor (PAF), cyclic phosphatidic acid, in addition to sphingomyelin [7,8,9].

Molecular biology techniques enabled the identification and heterologous expression of several recombinant PLDs isoforms from *Loxosceles* spiders’ venoms. These isoforms were characterized and contributed to unveil their participation on the pathophysiology of envenoming. *L. laeta* venom gland transcriptome showed that 16.3% of the ESTs encode PLDs [10]. Two PLDs from *L. laeta*, which were named SMase I and SMase II, were recombinantly obtained, and both displayed sphingomyelinase and complement-dependent hemolytic activities, in addition to triggering dermonecrosis in rabbit’s skin [11,12]; furthermore, polyclonal antibodies raised against SMase I efficiently neutralized its dermonecrotic activity after toxin and serum were incubated prior to injection in rabbits [11]. Machado and colleagues [13] performed proteomic analysis of *L. intermedia*, *L. laeta,* and *L. gaucho* crude venoms by using two-dimensional electrophoresis, revealing spots corresponding to PLDs (30 to 35 kDa); these spots were further analyzed through a combination of mass spectrometry, immunoblotting and chromatography, which resulted in the identification of 11 *L. gaucho* PLDs isoforms. LgRec1, a *L. gaucho* PLD recombinantly obtained, elicited platelet aggregation, induced sphingomyelinase cleavage and prompted direct hemolytic activity; LgRec1 also provoked local reactions near the injection site (i.e., edema and erythema) and dermonecrosis in rabbit’s skin, which were neutralized when LgRec1 was previously incubated with arachnidic antivenom, anti-*L. gaucho,* or anti-LgRec1 sera [14].

Structural analyses identified PLDs amino acid residues considered important for catalysis, substrate binding, and metal ion binding [15,16,17,18,19]. The mechanisms by which these PLDs interact and cleave their substrates have been described and are related to two theoretical models under discussion, which are the cleavage of phospholipids by hydrolysis or by transphosphatidylation [2,9,15]. All of this information is relevant for extending biotechnological applications, such as the use of these molecules in the production of neutralizing antibodies for serum therapy or the diagnosis of loxoscelism [20,21,22].

In this work, we aimed to perform comparative biochemical and biological analyses of wild type and mutants of PLDs from *L. laeta* and *L. gaucho* venoms. Results herein obtained may provide rational parameters for the use of recombinant PLDs as antigens for the development of a second-generation anti-*Loxosceles* serum. In addition, we present relevant data for the production of other therapeutic inputs such as a protective vaccine to be used in areas where envenomation by *Loxosceles* spiders are epidemic.

## 2. Materials and Methods

### 2.1. Wild Type and Site-Directed Mutant PLDs

Wild type LlRecDT1 (GenBank: AY093599.1) and LgRecDT1 (GenBank: JX866729.1) constructions were obtained by using pET-14b plasmids as cloning vectors, according to the methods described by Chaim and colleagues [23]. Wild type constructions previously mentioned were used for the generation of site-directed mutations H12A-H47A, E32A-D34A, Y228A, and Y228A-Y229A-W230A. Initially, sense oligonucleotides responsible for introducing the intended mutations were designed using the QuikChange Primer Design tool (https://www.agilent.com/store/primerDesignProgram.jsp (accessed on 7 February 2021)) (Table 1). The mutated isoforms were produced using pET-14b wild type constructions as templates and the QuikChange^®^ Multi Site-Directed Mutagenesis Kit (Agilent, Santa Clara, CA, USA) following manufacturers’ instructions. For this, PCR reactions were performed in order to amplify the mutated sequences. Next, amplified sequences were incubated with the endonuclease DpnI, which recognizes and hydrolyzes methylated, non-mutated strands containing the 5′-Gm6ATC-3′ target sequence. All of the mutated constructions were then transformed into chemically competent *Escherichia coli* XL10-gold bacterial strain (Agilent, Santa Clara, CA, USA). The colonies were subjected to plasmid mini-preparations using the WizardPlus SV Minipreps DNA Purification Systems^®^ Kit (Promega, Madison, WI, USA), followed by sequencing reactions using the reagent BigDye^®^ Terminator v3.1 Cycle Sequencing Kit (Applied Biosystems, Warrington, UK). Each clone was sequenced using the T7 sense primer or the T7 terminator primer by means of automated sequencing in the Applied Biosystems 3500 Genetic Analyzer (Applied Biosystems, Warrington, UK). The sequencing reactions were analyzed using ChromasPro (Version 2.6.4, Technelysium Pty Ltd., Brisbane, QLD, Australia) software to confirm the mutations produced. Comparison between wild type and mutated sequences was carried out using the ClustalW tool (https://www.genome.jp/tools-bin/clustalw (accessed on 7 February 2021)).

### 2.2. Recombinant Expression

The constructions containing the site-directed mutations were transformed into *Escherichia coli* BL21(DE3)pLysS and plated on Luria–Bertani (LB) agar plates supplemented with ampicillin (100 µg/mL) and chloramphenicol (34 µg/mL). An isolated colony was inoculated into 10 mL of liquid LB broth containing the antibiotics mentioned above at 37 °C for 16 h. Subsequently, the 10-mL culture was inoculated in 1 L of liquid LB containing the antibiotics at 37 °C and the culture growth was monitored until the optical density (OD) at 550 nm reached between 0.4 and 0.6. The isopropyl β-D-thiogalactoside (IPTG) inducer was then added to a final concentration of 0.05 mM and the culture was allowed to grow for additional 3.5 h at 30 °C, under vigorous shaking. Bacterial cells were finally harvested by centrifugation (3500× *g*, 10 min) and the resultant pellet was resuspended in 20 mL of binding buffer (50 mM NaH_2_PO_4_/Na_2_HPO_4_ pH 8.0, 500 mM NaCl, 10 mM imidazole). After adding lysozyme (1 mg/mL), the suspension was stored at −20 °C [23,24].

### 2.3. Protein Purification

The frozen resuspension was thawed, subjected to mechanical lysis using a French pressure cell press (3 cycles of 5 min on ice-pressure within 5000 and 10,000 psi) and centrifuged (9000× *g*, 30 min at 4 °C). The resulting supernatant was collected and 2 mL of Ni^2+^-NTA agarose resin was added to it, followed by incubation under gentle agitation for 1 h at 4 °C. The batch was exhaustively washed with washing buffer (50 mM NaH_2_PO_4_/Na_2_HPO_4_ pH 8.0, 500 mM NaCl, 20 mM imidazole) until the OD at 280 nm reached 0.01/0 or until the readings remained constant. The recombinant protein was eluted using elution buffer (50 mM NaH_2_PO_4_/Na_2_HPO_4_ pH 8.0, 500 mM NaCl, 250 mM imidazole), dialyzed against phosphate-buffered saline (PBS: 100 mM NaCl, 80 mM Na_2_HPO_4_, 20 mM NaH_2_PO_4_, pH 7.4) and the purity the eluate was analyzed by 12.5% SDS-PAGE under reducing conditions (β-mercaptoethanol 5%) [24].

### 2.4. Circular Dichroism

Circular dichroism spectra were acquired to compare the structure of the recombinant wild type proteins and their mutants. The purified proteins were dialyzed against phosphate buffer (20 mM NaH_2_PO_4_/Na_2_HPO_4_ pH 7.4 and 150 mM NaCl) at 4 °C and then diluted in the same buffer to a final concentration of 0.5 mg/mL. The spectra were acquired in a Jasco J-810 spectropolarimeter (Jasco Corporation, Tokyo, Japan) using a 1 mm path-length cuvette. Each spectrum, recorded at 0.5 nm intervals, consists of an average of 8 measurements performed at a rate of 50 nm/min, with a response time of 8 s and a bandwidth of 1 nm. The temperature was maintained constant at 20 °C [24,25]. Measurements were performed in triplicate. Secondary structures of the recombinant proteins were predicted using the online tool K2D3 (http://cbdm-01.zdv.uni-mainz.de/~andrade/k2d3 accessed on 7 February 2021).

### 2.5. Immunoassays

The interaction between wild type isoforms and mutated toxins with antibodies raised against *L. gaucho* or *L. laeta* crude venoms produced in rabbits were performed by both western blot and ELISA immunoassays. Initially, protein quantification was performed according to Bradford [26]. For the western blotting analysis, samples containing 2.5 µg of each protein were submitted to SDS-PAGE under reducing conditions and transferred onto a nitrocellulose membrane using the Trans-Blot^®^ SD Semi-Dry Electrophoretic Transfer Cell System (Bio-Rad, Hercules, CA, USA) for 30 min at 25 V. The membranes were incubated with hyperimmune sera raised against *L. gaucho* or *L. laeta* crude venom (1:3000). Control of antivenom sera specificity was performed using pre-immune serum. After incubation with the mentioned sera, membranes were treated with secondary alkaline phosphatase-conjugated anti-IgG antibody (1:5000) (Sigma-Aldrich, St. Louis, MO, USA). Finally, results were developed using BCIP/NBT substrate reaction (Promega, Madison, WI, USA). For the ELISA antibody capture assay, a 96-well microplate (Nunc MaxiSorp, Roskilde, Denmark) was coated with a solution containing 1 μg/mL protein (100 μL per well) in 0.02 M sodium bicarbonate buffer pH 9.6 overnight at 4 °C. After that, the plate was washed with PBS-Tween 20 solution (0.05%), blocked with PBS-casein (2%) for 1 h at 37 °C and the reactions were incubated for 1 h with hyperimmune sera against *L. gaucho* or *L. laeta* crude venom (1:10,000) at 37 °C. Control of antivenom sera specificity was performed with pre-immune serum. Thereafter, the plate was washed with PBS-Tween 20 solution (0.05%) and wells were incubated with secondary antibodies against IgG-rabbit conjugated with horseradish peroxidase (Sigma Aldrich, San Luis, MO, USA) (1:5000) for 1 h at 37 °C. Finally, the colorimetric reactions were developed using a solution containing 0.4 mg/mL ortho-phenylene diamine (OPD) with 4 μL/mL H_2_O_2_ in citrate buffer for peroxidase pH 5.0 (50 mM NaH_2_PO_4_, 24 mM citric acid) for 30 min at 25 °C and reactions were then stopped with 50 μL of 1 M H_2_SO_4_. Absorbance values were measured at 490 nm in spectrophotometer (Meridian ELX 800) [23,27]. Each treatment was assessed in quadruplicate and the results are shown as mean ± SEM of three independent experiments.

### 2.6. Structural Analyses

The analysis of the three-dimensional structures was performed using the crystallographic structures of the PLDs of *L. intermedia* (Protein Data Bank (PDB) ID: 3RLH) and *L. laeta* (PDB ID: 2FR9), and the structural models of the mutants built using SWISS-MODEL based on their respective PDB IDs as a template. The native and mutants *L. gaucho* PLDs models were constructed using the *L. intermedia* PDB ID 3RLH as a template. The electrostatic potential was calculated using the program APBS according to the molecular electrostatic potential ranging from −5.0 to 5.0 [28] and implemented in PYMOL (version 2.4, GitHub, San Francisco, CA, USA) [29].

### 2.7. Sphingomyelinase Activity

Sphingomyelinase activity was determined using the Amplex Red Assay Kit (Thermo Fisher Scientific, Waltham, MA, USA). To measure the sphingomyelinase activity of the recombinant toxins, they were incubated (10 μg) in microtubes containing the Amplex Red reagent mixture in reaction buffer (Tris-HCl 100 mM pH 7.4 containing 10 mM MgCl_2_), including the substrate sphingomyelin (SM: egg sphingomyelin, chicken). Wild type recombinant isoforms, LlRecDT1 and LgRecDT1, were used as positive controls and reactions containing only the Amplex Red reagent with the substrate SM (without any toxin) were used as negative controls. After incubation at 37 °C for 30 min, the reactions were transferred from the microtubes to a 96-well black fluorimeter plate and the fluorescence was measured on a fluorimeter (Tecan Infinite M200, Männedorf, Switzerland) using excitation wavelength at 540 nm and emission at 570 nm. All treatments were performed in triplicate and the results represent an average of three experiments ± SEM.

### 2.8. High Performance Thin Layer Chromatography (HPTLC)

The phospholipase activity of the recombinant PLDs was also assessed by HPTLC. For this, samples of 50 μg of each PLD were incubated with 1 mg/mL of sphingomyelin (SM) for 2 h at 37 °C. The organic phase was recovered using 1 mL of a water:butanol solution (1:1), from which 200 μL was collected and dried. Next, the organic phase was resuspended in chloroform and 20 μL was then applied to the base of the thin-layer chromatographic silica plate with capillary tubes. A solution containing chloroform:methanol:methylamine (65:35:10, *v*/*v*/*v*) was used as the mobile phase [24]. The samples were visualized as blue spots when the plates were sprayed with molybdenum blue dye. The results shown are from one experiment out of two independent experiments that were performed.

Densitometry of the digital images from the HPTLC plates were acquired using the GeneSnap software for G: Box Chemi XL (Syngene, Cambridge, UK) and quantified by the Quantity One software for Chemic Doc XRS (Bio-Rad, Hercules, CA, USA). The percentages of cleavage by LlRecDT1 and LgRecDT1 were considered as 100%. Results represent the densitometry of one experiment.

### 2.9. Animals

Adult Swiss mice weighing between 25 and 30 g were supplied by the Animal House of the Biological Sciences Center of the Federal University of Paraná and adult New Zealand rabbits weighing approximately 3 kg were acquired from the Canguiri Experimental Farm of Federal University of Parana and kept at Production and Research Center of Immunobiological Products (CPPI, Brazil). Animal experiments were carried out in accordance to the Brazilian Guidelines for Care and Use of Animals for Scientific and Teaching Purposes established by the National Council for Control of Animal Experimentation (CONCEA) and the International Guidelines for animal experimentation. The Ethics Committee for Animal Use from the Biological Sciences Sector of the Federal University of Parana (CEUA/BIO-UFPR) approved all of the protocols using animals in this work (Statement number 1112).

### 2.10. Hemolytic Activity

The hemolysis assay was conducted as described by Chaves-Moreira and colleagues [7], with modifications. Initially, 4 mL of rabbit blood was harvested with acidic EDTA Na^+^ 5% and centrifuged at 2000× *g* for 15 min, followed by aspiration and discard of the overlying platelet-rich plasma and buffy coat. The remaining erythrocytes were washed three times in Tris buffer sucrose (TBS: 250 mM sucrose, 10 mM Tris/HCl, pH 7.4). After washing steps, the erythrocytes were resuspended in TBS with 1 mM CaCl_2_ to obtain an initial concentration of 5 × 10^8^ cells/mL. Aliquots containing 200 μL of the erythrocyte suspension were placed in microtubes, followed by addition of 200 μL of a solution containing wild type or mutant PLDs (10 μg) solubilized in TBS. Negative control reactions contained only cell suspensions just in TBS and positive control reactions were constituted by cell suspensions in distilled water and Triton X-100 0.1% (*v*/*v*). The reactions were incubated under gentle and constant agitation at 37 °C. After the treatment times (0, 3, 6, 12, and 24 h), the microtubes were centrifuged at 4 °C using a refrigerated microfuge (Centrifuge 5804 R, Eppendorf, Hamburg, Germany), for 3 min at 3500× *g*. The absorbance of free hemoglobin in the supernatant was read at 550 nm in a spectrophotometer (Meridian ELx 800, BioTek Instruments, Winooski, VT, USA). Absorbance readings were converted to percent hemolysis considering the positive control absorbance as 100% lysis. Each treatment was assessed in triplicate and the results are shown as mean ± SEM of three independent experiments performed.

### 2.11. Vascular Permeability Assay

In order to evaluate alterations triggered by the recombinant PLDs in the microvascular permeability, the Miles assay was performed [24,30]. For this analysis, mice were intravenously injected with Evans Blue diluted in PBS (30 mg/kg), followed by intradermal administration of wild type or recombinant mutant PLDs samples (10 μg) 5 min later at the dorsolateral region of the animals. Each treatment group consisted of five animals and the negative control was represented by animals that received the vehicle (PBS) without any toxin. After 1 h, the mice were euthanized using ketamine (30 mg/kg) and xylazine (4 mg/kg) and the dye leakage was visualized (bluish stain). A tissue patch of the dorsal skin from each animal was removed and photographed.

### 2.12. In Vivo Dermonecrosis

PLDs dermonecrotic activity was evaluated after intradermal application of 10 μg of the wild type and recombinant mutant toxins at a dorsolateral shaven area of rabbits’ skin. PBS solution was administered to animals of the control group. The test was performed in duplicate for each sample. Animals were monitored throughout the evolution of the dermonecrotic reaction and macroscopic images were taken after 0, 3, 6, and 24 h post toxin injection. After the maximum exposure time (24 h), the rabbits were euthanized with ketamine (240 mg/kg) and xylazine (28 mg/kg), followed by tissue excision for histopathological analysis purpose [24].

### 2.13. Histological Methods for Light Microscopy

Excised rabbit skin sections were fixed in Alcohol–Formalin–Acetic Acid (ALFAC) fixative solution (ethanol 85%, formaldehyde 10%, and glacial acetic acid 5%) for 16 h at 4 °C, dehydrated in a graded series of ethanol and embedded in paraffin for 2 h at 58 °C. The fixed samples were processed in thin sections (4 μm), placed on glass slides for histological analysis, and stained with hematoxylin and eosin (H&E). The sections were analyzed and photographed under a light microscope Olympus BX41 with DP 72 camera for image capture (Olympus, Tokyo, Japan).

### 2.14. Statistical Analyses

Values were expressed as mean and standard error of the mean. Statistical analysis of the ELISA and hemolytic assays were performed using two-way analysis of variance (ANOVA) followed by post-hoc Tukey test for average comparisons using the GraphPad Prism 6 program (GraphPad Software, San Diego, CA, USA), considering *p* value < 0.001 as statistically significant. Statistical analysis of the Sphingomyelinase activity assay was performed using one-way analysis of variance (ANOVA) followed by post-hoc Tukey test for average comparisons using the GraphPad Prism 6 program, considering *p* value < 0.05 as statistically significant.

## 3. Results

### 3.1. Analysis of Sequences Encoding Wild type PLDs, Site-Directed Mutagenesis, and Production of Mutant PLDs Isoforms

**Figure 1:**
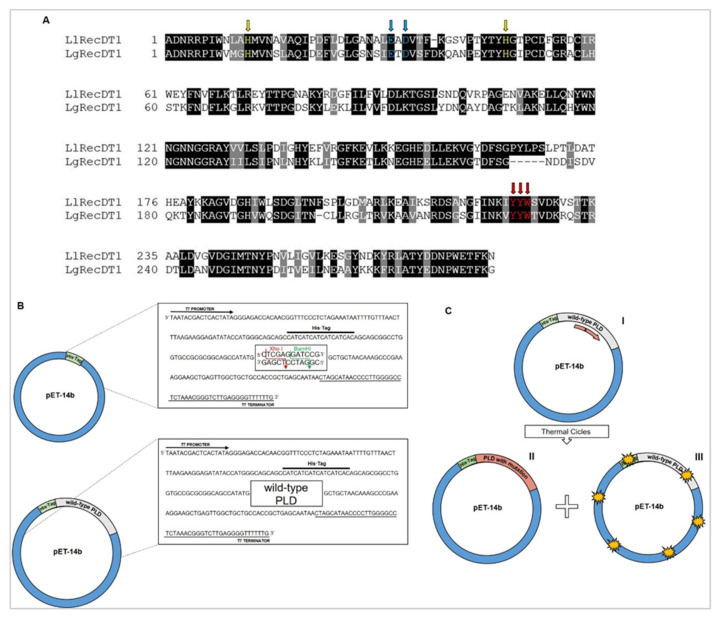
Overview of the wild type and mutant Phospholipases-D (PLDs)-sequence alignment and scheme of the constructions produced for heterologous expression. (**A**) Alignment of the cDNA-deduced amino acid sequences encoding the wild type PLDs of *L. gaucho* (LgRec1—GenBank: JX866729.1) and *L. laeta* (SMase I—GenBank: AY093599.1). Box shade representations show the conserved amino acid residues shaded in black and conservative substitutions are highlighted in gray. Yellow arrows indicate the sites of H12A-H47A mutations; blue arrows show the E32A-D34A mutation, only for *L. gaucho*; red arrows point out the site of both mutagenesis Y228A and Y228A-Y229A-W230A; (**B**) schematic representations of pET-14b vector (Promega) displaying the restriction sites for XhoI and BamHI enzymes and the N-terminal 6× His-tag, and the construction containing the wild type PLD sequence cloned in the vector; (**C**) schematic representation of the steps followed to obtain the constructions containing the sequence encoding mutant PLDs. The red arrow represents the mutagenic primer annealing with the wild type PLD coding sequence (I); after thermal cycles, the enzyme blend closes the nicks generating a construct with the specific mutation (II); and, prior to transformation in BL21(DE3)pLysS *E. coli*, the DpnI enzyme digests the methylated remaining wild type templates (III).

The cDNA-deduced amino acid sequences encoding the wild type PLDs of *L. laeta* (LlRecDT1) and *L. gaucho* (LgRecDT1) revealed 285 and 280 amino acid residues, respectively, while the degree of sequence identity between these PLDs is 56.84% (Figure 1A). In addition, the two sequences exhibit the conservation of important amino acid residues, which were identified by crystallographic findings [15,31] (Figure 1A). Therefore, specific primers (Table 1) allowed the obtainment of mutants regarding important amino acid residues important for the enzyme structure and that are involved in the functionality of the PLDs: LlRecDT1 H12A-H47A and LgRecDT1 H12A-H47A mutants produced contain mutations in amino acid residues essential for the PLD’s catalytic activity (H12 and H47). LgRecDT1 E32A-D34A mutant obtained display mutations in amino acid residues required for the coordination of the magnesium ion (E32 and D34). LlRecDT1 Y228A and LgRecDT1 Y228A, as well as LlRecDT1 Y228A-Y229A-W230A and LgRecDT1 Y228A-Y229A-W230A mutants, were generated with mutations associated with three amino acids residues containing aromatic side chains and which are located at the PLD’s binding site to lipid substrates (Y228, Y229, and W230) [2,15,20,31]. All selected amino acid residues were replaced by an alanine residue. The wild type and mutated PLDs sequences were cloned in pET-14b vector and sequencing analysis confirmed the constructions (Figure 1B,C). These constructions were then transformed into BL21(DE3)pLysS *E. coli* competent cells.

### 3.2. Expression and Purification of Recombinant PLD Wild type and Mutant Isoforms

**Figure 2:**
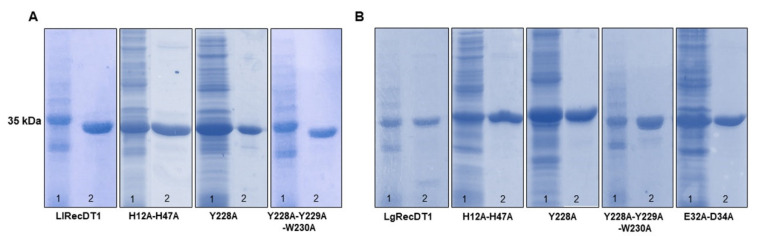
PLDs recombinant expression and protein purification analyzed by 12.5% SDS-PAGE under reducing conditions and stained with Coomassie blue dye. Lanes numbered as 1 represent samples after expression in *E. coli* BL21 (DE3)pLysS cells for 3.5 h at 30 °C with 0.05 mM isopropyl β-D-thiogalactoside (IPTG). Lanes numbered as two represent samples of purified PLDs (5 µg) after purification by Ni-NTA affinity chromatography: (**A**) *L. laeta* PLDs—LlRecDT1 and its mutants; (**B**) *L. gaucho* PLDs—LgRecDT1 and its mutants.

Wild type and mutated PLDs were expressed in *E. coli* BL21 (DE3)pLysS and purified as soluble products using affinity chromatography (Figure 2A,B). The yield of all recombinant proteins ranged from 6 to 12 mg/L. Attempts to obtain the mutant isoform LlRecDT1 E32A-D34A were unsuccessful, resulting in aggregation and consequent production of an insoluble product (data not shown). As shown in Figure 2A,B, all expressed recombinant isoforms displayed molecular masses of approximately 35 kDa as predicted by theoretical analysis and described in the literature [11,14].

### 3.3. Circular Dichroism Spectroscopy Analysis

**Figure 3:**
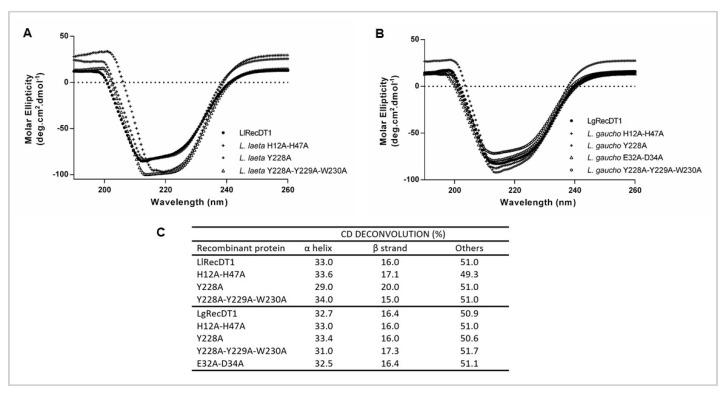
PLDs secondary structures analyzed by circular dichroism (CD). CD spectra were acquired using proteins samples at a concentration of 0.5 mg/mL in phosphate buffer solution, pH 7.4 at 20 °C: (**A**) LlRecDT1 and its mutants; (**B**) LgRecDT1 and its mutants; (**C**) spectra deconvolution showing the percentages of each secondary structure in the recombinant PLDs.

In order to verify the secondary structure profiles and the overall folding of the recombinant toxins produced, normalized solutions of *L. laeta* and *L. gaucho* purified recombinant PLDs were analyzed by circular dichroism (CD) spectroscopy, followed by deconvolution analysis of the generated spectra. As shown in Figure 3A,B, the spectra obtained and deconvolution data indicated similar proportions of alpha-helices, beta-sheets, and other structures for all recombinant PLDs, pointing out that regardless the incorporated mutations, the mutant toxins maintain their native-like conformation when compared to their respective wild type isoforms [24].

### 3.4. Molecular Modeling Analysis

**Figure 4:**
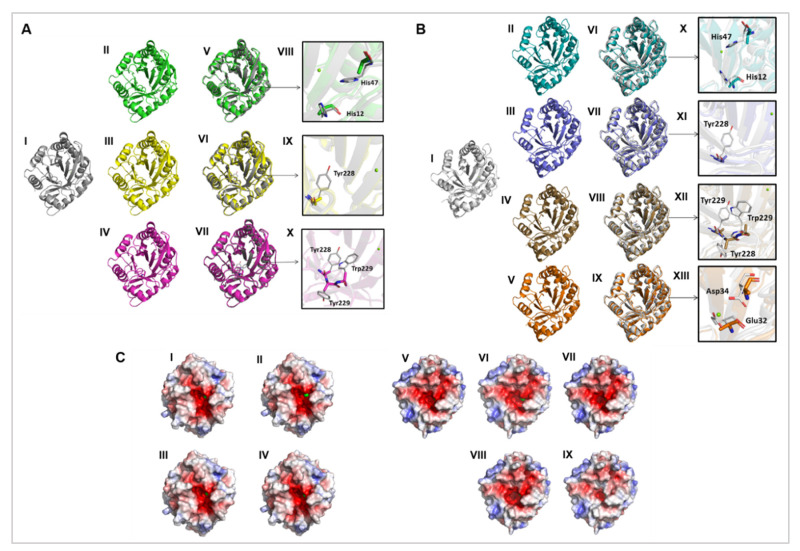
(**A**) Cartoon representation of the crystal structure of native PLD of *L. laeta* and molecular models of *L. laeta* mutants. The magnesium ion is represented by green spheres. (I) Crystal structure of the native *L. laeta* PLD in gray (Protein Data Bank ID: 2FR9); (II) structural model of *L. laeta* H12A-H47A in green; (III) structural model of *L. laeta* Y228A in yellow; (IV) structural model of *L. laeta* Y228A-Y229A-W230A in pink; (V) superposition of I and II; (VI) superposition of I and III; (VII) superposition of I and IV; (VIII) zoom on mutated residues with native H12 and H47 represented by lines with gray side chain and mutated A12 and A47 represented by sticks with green side chain; (IX) zoom on mutated residues with native Y228 represented by lines with gray side chain and mutated A228 represented by sticks with yellow side chain; (X) zoom on mutated residues with native Y228-Y229-W230 represented by lines with gray side chain and mutated A228-A229-A230 represented by sticks with pink side chain; (**B**) cartoon representation of the molecular model of native and mutant *L. gaucho* PLD. The magnesium ion is represented by green spheres. (I) Structural model of native *L. gaucho* PLD in gray; (II) structural model of *L. gaucho* H12A-H47A in cyan; (III) structural model of *L. gaucho* Y228A in blue; (IV) structural model of *L. gaucho* Y228A-Y229A-W230A in brown; (V) structural model of *L. gaucho* E32A- D34A in orange; (VI) superposition of I and II; (VII) superposition of I and III; (VIII) superposition of I and IV; (IX) superposition of I and V; (X) zoom on mutated residues with native H12 and H47 represented by lines with gray side chain and mutated A12 and A47 represented by sticks with cyan side chain; (XI) zoom on mutated residues with native Y228 represented by lines with gray side chain and mutated A228 represented by sticks with blue side chain; (XII) zoom on mutated residues with native Y228-Y229-W230 represented by lines with gray side chain and mutated A228-A229-A230 represented by sticks with brown side chain; (XIII) zoom on mutated residues with native E32-D34 represented by lines with gray side chain and mutated A32-A34 represented by sticks with orange side chain; (**C**) electrostatic potential visualization of *Loxosceles laeta* and *L. gaucho* PLDs. Surface charge distribution around the surface of *L. laeta* native PLD (I) and its mutants H12A-H47A (II), Y228A (III), Y228A-Y229A-W230A (IV). The potentials red, white, and blue represent negative, neutral and positive charges, respectively. Electrostatic potential visualization of *L. gaucho* PLDs. Surface charge distribution around the surface of *L. gaucho* native PLD (V) and its mutants H12A-H47A (VI), Y228A (VII), Y228A-Y229A-W230A (VIII), and E32A-E34A (IX). The potentials red, white, and blue represent negative, neutral, and positive charges, respectively.

Using as parameters *L. intermedia* and *L. laeta* PLDs crystallography data available [15,31,32], a molecular modeling analysis was performed to compare electrostatic potentials and solvent accessible surface areas between wild type PLDs and their mutants (Figure 4). Global Model Quality Estimation score and Qualitative Model Energy Analysis scores presented values, which indicated high reliability in the generated models. Molecular modeling examination did not show significant differences in the topographic organization (Figure 4A,B) and charges densities (Figure 4C) when mutant PLDs were compared to their wild type counterparts. Together with CD data, these results reinforce that the mutations did not cause substantial structure changes.

### 3.5. Immunological Cross-Reactivity between Recombinant PLDs and Native Venom Toxins

**Figure 5:**
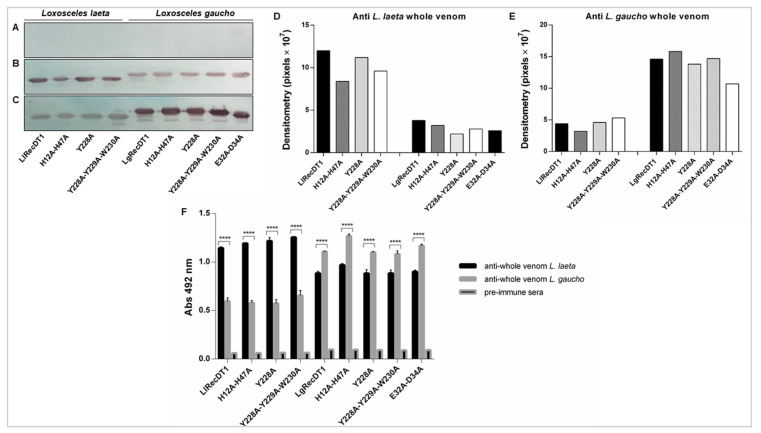
Immunoassays analyses—western blotting (WB) and ELISA. For WB analysis, samples of each purified PLD (2.5 μg) were separated by 12.5% SDS-PAGE under reducing conditions, then transferred onto nitrocellulose membranes that were exposed to: (**A**) pre-immune serum or (**B**) hyperimmune serum raised against *L. laeta* crude venom or (**C**) hyperimmune serum raised against *L. gaucho* crude venom. All sera were obtained from rabbits and used at a 1:1000 dilution; (**D**) densitometry of the bands obtained in the WB showing reactivity between LlRecDT1 and its mutants with hyperimmune serum raised against *L. laeta* venom as well as cross-reactivity between LgRecDT1 and its mutants with the same serum; (**E**) densitometry of the bands obtained in the WB showing reactivity between LgRecDT1 and its mutants with hyperimmune serum raised against *L. gaucho* venom as well as cross-reactivity between LlRecDT1 and its mutants with the same serum; (**F**) antibody-capture ELISA assay was performed using samples of each purified PLD (1 µg/mL) that were immobilized in the microplate wells and incubation with pre-immune and hyperimmune raised against *L. gaucho* and *L. laeta* crude venom obtained from rabbits at a 1:10,000 dilution. Each treatment was assessed in quadruplicate. The values are shown as averages ± SEM (standard error of the mean) of three independent experiments, with significance level at **** *p* < 0.0001 comparing pre-immune with antivenom sera raised against *L. laeta* and *L. gaucho* crude venom.

To examine the immunological cross-reactivity among antibodies against native venom toxins and recombinant PLDs, western blotting (WB), and ELISA assays were carried out. WB was performed to detect linear and denaturation-resistant epitopes while ELISA was conducted to identify conformational and non-denatured epitopes. Pre-immune serum in WB did not react with the recombinant PLDs (Figure 5A). In contrast, hyperimmune serum against *L. laeta* crude venom reacted with all of the wild type isoforms and their respective mutant isoforms (Figure 5B). The same findings were observed when a hyperimmune serum against *L. gaucho* crude venom was used (Figure 5C). The densitometry of the WB bands showed that, although the serum to *L. laeta* venom toxins cross-reacted with *L. gaucho* recombinant PLDs, the recognition of *L. laeta* recombinant toxins were qualitatively greater; serum against *L. gaucho* venom toxins also recognized *L. gaucho* recombinant PLDs in a greater extent when compared to *L. laeta* PLDs (Figure 5D,E). ELISA demonstrated that the recombinant proteins were recognized by the polyclonal sera raised against the crude venoms of *L. laeta* and *L. gaucho*, pointing out the epitopes’ conservation among native toxins and recombinant isoforms (Figure 5F). The serum against the crude venom of *L. gaucho* recognized all of the recombinant proteins of *L. laeta*. Similarly, all of the recombinant proteins of *L. gaucho* were recognized by the serum raised against the crude venom of *L. laeta.* However, it is well evidenced that the recognition of recombinant proteins from each species was quantitatively higher when the serum raised against the crude venom of the same species was used (Figure 5F).

### 3.6. Sphingomyelinase Activity

**Figure 6:**
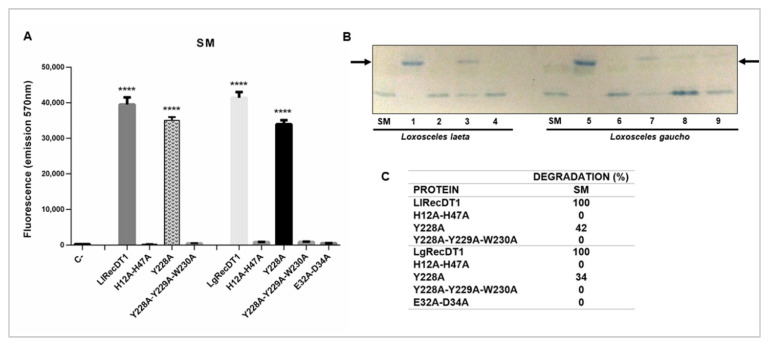
Comparative sphingomyelinase activity between the wild type PLDs (LgRecDT1 and LlRecDT1) and their mutant isoforms. (**A**) Amplex Red assay was performed at 37 °C for 1 h using 10 µg of each toxin and sphingomyelin (substrate). The fluorescent reaction product (resorufin) was detected on a fluorimeter using excitation wavelength at 540 nm and emission at 570 nm. LlRecDT1 and LgRecDT1 (wild type recombinant PLDs) were used as positive controls and reactions without any toxin were used as negative controls. The values represent averages of three experiments (*n* = 5) ± SEM and values with **** *p* < 0.05 were considered statistically significant; (**B**) HPTLC analysis was carried out using 50 μg of each PLD and 1 mg/mL of sphingomyelin (SM) as substrate (representative image of two independent experiments). Reactions containing sphingomyelin without any proteins were used as negative control. The lipids were extracted with butanol and applied to the plate for separation, followed by the visualization of bands stained with molybdenum blue. Arrows show bands corresponding to sphingomyelin cleavage products following protein treatments. Numbers shown in the figure represent: (1) LlRecDT1; (2) LlRecDT1 H12A-H47A; (3) LlRecDT1 Y228A; (4) LlRecDT1 Y228A-Y229A-W230A; (5) LgRecDT1; (6) LgRecDT1 H12A-H47A; (7) LgRecDT1 Y228A; (8) LgRecDT1 Y228A-Y229A-W230A; (9) LgRecDT1 E32A-D34A; (**C**) densitometry of the bands corresponding to sphingomyelin cleavage products after incubation with the recombinant PLDs. Quantification was performed in one HPTLC experiment. The percentage of cleavage by the wild type recombinant PLDs (LlRecDT1 and LgRecDT1) were considered as 100%.

Two experimental approaches were carried out in order to evaluate the sphingomyelinase activity of the wild type and mutants PLDs: spectrofluorimetric quantification of enzymatic products and detection of degradation products by HPTLC. As shown in Figure 6, the sphingomyelin-cleavage activity of isoforms with mutations in the catalytic site (H12A-H47A) or in the magnesium ion coordinating region (E32A-D34A), as well as the isoforms containing the triple mutation of aromatic amino acid residues (Y228A-Y229A-W230) displayed only residual activity in both experiments when compared to the wild type controls (LlRecDT1 or LgRecDT1). Interestingly, the Y228A mutation did not give rise to inactive isoforms, as shown by the slight decrease on sphingomyelin cleavage on the Amplex Red assay and the maintenance of 42% and 34% percentage of sphingomyelinase cleavage for LlRecDT1 Y228A and LgRecDT1 Y228A, respectively. The difference in the cleavage activity between the assays reflects the specific sensitivity of the two methods used, but indicates that Y228A mutation did not abolish sphingomyelin-cleavage activity on the mutant PLDs.

### 3.7. Hemolytic Activity

**Figure 7:**
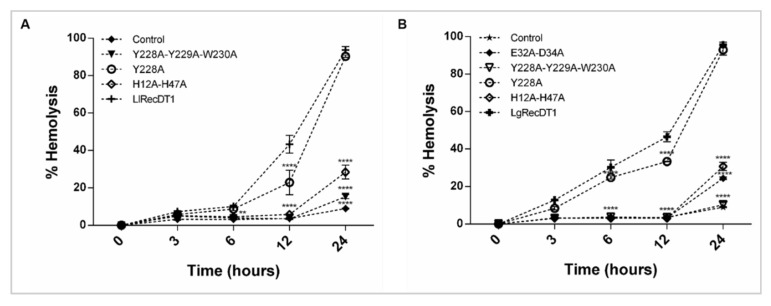
Hemolytic activity of recombinant PLDs on rabbit erythrocytes. PLDs samples (10 µg) in TBS were incubated with erythrocytes for 0, 3, 6, 12, and 24 h at 37 °C. Reactions containing cell suspensions without any toxin and reactions containing cell suspensions incubated with 0.1% Triton X-100 were used as negative and positive controls, respectively. The absorbance of supernatants was measured at 550 nm and the percentage of hemolysis was determined considering the absorbance values of the positive control as 100%. The values represent the averages of three experiments ± SEM and values with **** *p* < 0.001 were considered statistically significant; (**A**) hemolysis percentages for LlRecDT1 and its mutant isoforms; (**B**) hemolysis percentages for LgRecDT1 and its mutant isoforms.

The cytotoxic effects of recombinant toxins resulting in hemolysis on red blood cells were assessed according to previous works, which have shown that brown spider PLDs are hemolytic [7,33]. As shown in Figure 7, the wild type recombinant toxins from the two venoms exhibited high hemolytic activity, resulting in hemolysis percentages close to 100% in the 24 h treatment. Both Y228A-mutation isoforms presented slightly reduced hemolytic activity when compared to the wild type recombinant PLDs. H12A-H47A, Y228A-Y229A-W230A, and E32A-D34A mutations led to a massive inhibition of the hemolytic activity when compared to their wild type counterparts. The observed hemolytic activity was time-dependent for both *L. laeta* and *L. gaucho* PLDs (Figure 7).

### 3.8. Activity on Blood Vessel Permeability

**Figure 8:**
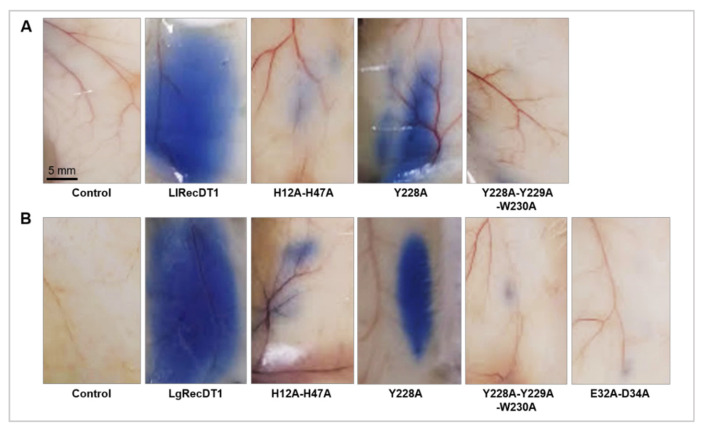
Vascular permeability assay. Mice (*n* = 5 for each treatment) were administered intravenously with Evans Blue (EB) dye (30 mg/kg) followed by intradermal injection of PLDs samples (10 μg) diluted in PBS 5 min later. Dorsal skin tissue samples were removed after 1 h in order to visualize EB dye extravasation. PBS was used as negative control, corresponding to regular permeability level: (**A**) photographs show EB leakage after treatment with *L. laeta* wild type PLD and its mutant isoforms; (**B**) photographs show EB leakage after treatment with *L. gaucho* wild type PLD and its mutant isoforms.

In order to evaluate the activity of the PLDs on blood vessels properties, the Miles Assay was performed. As depicted in Figure 8, both wild type recombinant toxins caused a diffuse Evans Blue dye leakage to the extracellular compartment when compared to the PBS treatment (negative control). H12A-H47A, Y228A-Y229A-W230A, and E32A-D34A mutations resulted in residual capillary permeability, pointing out that these mutant PLDs did not induce severe alterations in the structure and functionality of blood vessel walls. In contrast, the Y228A mutated PLD was able to increase capillary permeability (Figure 8).

### 3.9. Dermonecrotic Activity

**Figure 9:**
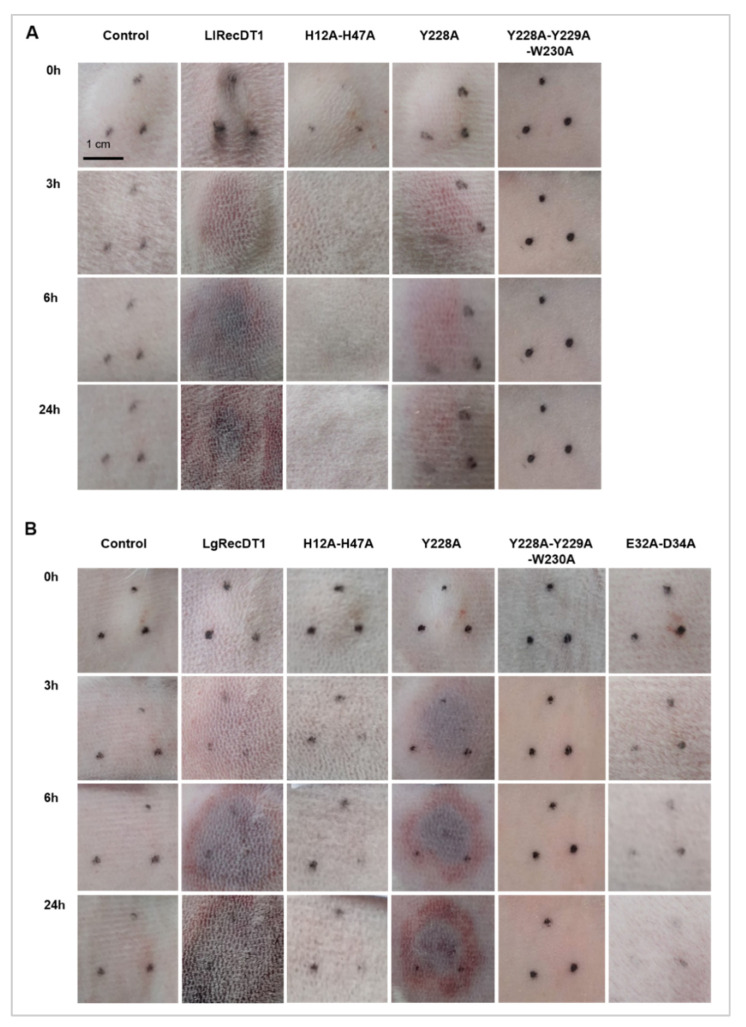
Macroscopic analysis of dermonecrotic lesions in rabbits after treatment with wild type PLDs and its mutant isoforms (10 μg). Macroscopic aspect of the lesions near the injection site was observed and photographed 0, 3, 6, and 24 h after treatments. PBS was used as negative control. Black dots show the local of the injection: (**A**) lesions induced by *L. laeta* PLDs; (**B**) lesions induced by *L. gaucho* PLDs.

The major sign of the cutaneous loxoscelism is the dermonecrotic lesion observed on the skin of injured patients, mainly triggered by the action of PLDs. To evaluate the necrotic lesion induced by the recombinant PLDs, toxins were injected on the skin of rabbits and the lesions were observed at a macroscopic level (0, 3, 6, and 24 h after treatments). Both wild type recombinant toxins provoked a range of local reactions near the injection site such as swelling, erythema, and tissue necrosis, which was detected by the darkening of the skin (Figure 9). H12A-H47A, Y228A-Y229A-W230A, and E32A-D34A mutations inhibited the assayed activity to macroscopically undetectable levels, similar to the negative control. The Y228A mutation, in its turn, was not able to inhibit the development of the lesion (Figure 9).

### 3.10. Histopathological Changes

**Figure 10:**
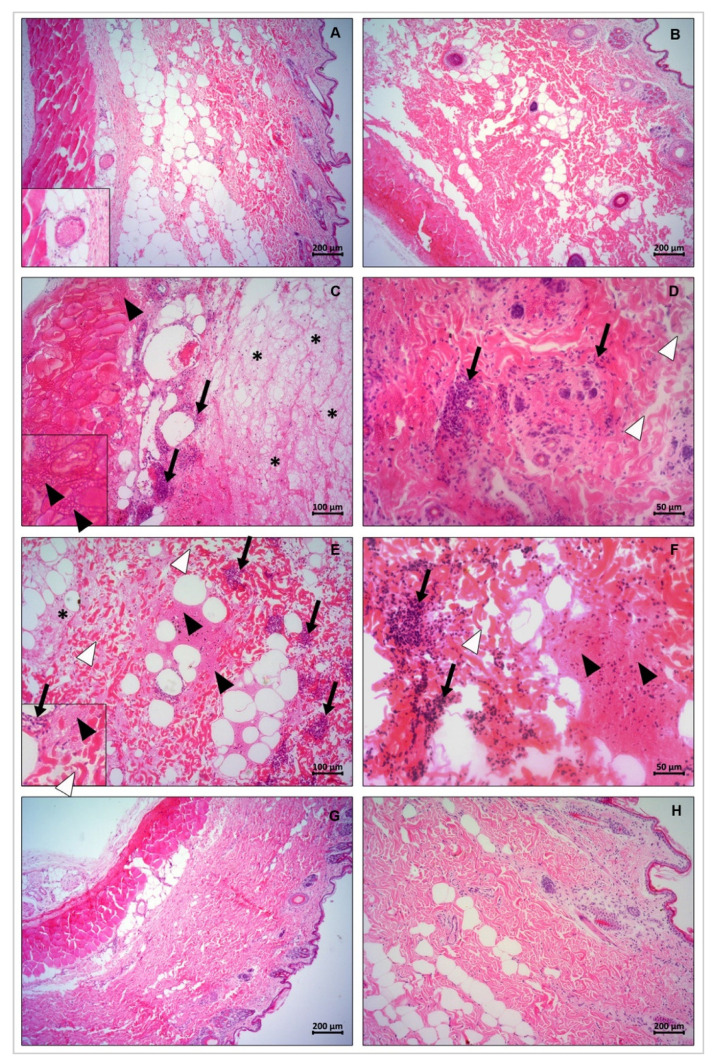
Histopathological analysis of the lesions in rabbits after treatment with wild type PLDs and its mutant isoforms (10 μg). Histological images of rabbits’ skin 24 h after injection of LlRecDT1, LgRecDT1, and their mutants: (**A**,**B**) normal aspect of the tissue after injection of PBS. In A, subfigure highlights an intact blood vessel (zoom in); (**C**,**D**) tissue damage after exposition to LgRecDT1 and LlRecDT1, respectively. Tissue impairment is observed by the presence of hemorrhage (black arrowheads), inflammatory cells (black arrows), disorganization of collagen fibers (white arrowheads), as well as fibrin network deposition (asterisks). In C, subfigure highlights the signs of hemorrhage (zoom in); (**E**) tissue aspect after injection with Y228A mutation of *L. gaucho*; disorganization of collagen fibers (white arrowheads), hemorrhage (black arrowheads), inflammatory cells (black arrows) and fibrin network deposition (asterisks) are present. Subfigure shows details in the disorganization of collagen fibers (white arrowheads), hemorrhage (black arrowheads) and inflammatory cells (black arrows) (zoom in); (**F**) inflammatory cells accumulation (black arrows), disorganization of collagen fibers (white arrowhead) and hemorrhage (black arrowheads) caused by Y228A mutation of *L. laeta*; (**G**,**H**) tissues with no effects after injection with E32A-D34A and H12A-H47A mutant PLDs of *L. gaucho*, respectively.

Skin sections excised from the areas of PLD injections were submitted to light microscopic analysis to evaluate histopathological alterations (Figure 10). Based on the acquired images, wild type recombinant PLDs (LlRecDT1 and LgRecDT1) induced a series of tissue impairments when compared to the negative control (PBS), such as disorganization of dermis collagen fibers, hemorrhage, fibrin network deposition, and presence of dense infiltrates of inflammatory cells in the dermis. Both LgRecDT1 Y228A and LlRecDT1 Y228A caused the same tissue alterations verified for the wild type recombinant PLDs, pointing out that the correspondent mutation did not generate a biologically inactive isoform. On the other hand, LgRecDT1 E32A-D34A and LgRecDT1 H12A-H47A did not trigger any noxious effects, which can be confirmed by the normal tissue structure observed in their respective histological sections; these results suggest that the E32A-D34A and H12A-H47A mutations generated biologically inactive PLDs (Figure 10).

## 4. Discussion

Accidents caused by brown spider bites constitute a public health problem in many parts of the world, especially in South American countries such as Brazil, Argentina, Mexico, Peru, and Chile. In these countries, *Loxosceles* spiders are endemic and accidents with them have resulted in critical problems to exposed populations. In the United States of America, especially in the South and Center-South States, many accidents are also reported. In Brazil, these accidents prevail mainly in populations living in the southern states [1,2,34,35,36,37]. The treatment currently established for loxoscelism is essentially symptomatic for the cutaneous form and it aims to halt the robust inflammatory response, which is a key process associated to the pathophysiology of the accidents, as well as the dermonecrosis (aseptic coagulative necrosis). Treatments include steroidal anti-inflammatory drugs such as prednisolone or dapsone, in addition to analgesics as acetylsalicylic acid or paracetamol. Antibiotics, such as erythromycin or cephalosporins, can also be used to treat secondary infections, and antihistamines have also been prescribed to reduce allergic reactions or skin rash [1,2,34,35,38,39,40]. In the case of systemic loxoscelism, also known as viscero-cutaneous form, treatments include steroidal anti-inflammatory drugs and antivenom serum therapy. In the most severe cases, antivenom serum therapy is widely adopted in countries such as Brazil, Argentina, Mexico, and Peru [1,2,34,35,38,39,40]. In some regions in Brazil, especially in the southeastern states, the antivenom serum therapy used involves the intravenous application of the polyvalent anti-arachnidic serum produced by the Butantan Institute in São Paulo. This anti-arachnidic serum is obtained by horse immunization with a mixture of the crude venoms from the spiders *Loxosceles gaucho* and *Phoneutria nigriventer*, as well as the crude venoms from the scorpions *Tityus serrulatus* and *Tityus bahiensis*. In Brazil’s southern states, the serum therapy is based on the administration of the anti-*Loxosceles* serum, which is produced by the Production and Research Center of Immunobiological Products (CPPI) in Paraná state. The anti-*Loxosceles* serum is also obtained by horse immunization with the crude venoms from the brown spiders *L. intermedia*, *L. laeta,* and *L. gaucho*, which are the most important species from the medical perspective in South America [1,2,34,35].

The use of antivenom sera in the treatment of loxoscelism is controversial and it is not a practice performed in the United States of America, for example. The use of anti-*Loxosceles* serum therapy in cases of the systemic loxoscelism has been successful, which is supported by experimental and clinical data. A study has already shown that the pediatric mortality decreased after serum therapy [1,41]. Besides that, it has also been shown that anti-*Loxosceles* serum experimentally administered in rabbits reduced the noxious alterations often seen followed venom exposure, such as hematological disorders, extravascular deposition of fibrinogen and alterations in renal function [1,42]. On the other hand, experimental and clinical evidence supports that antivenom serum therapy is ineffective in the treatment of cutaneous loxoscelism for reasons that still remain unknown [42]. It is speculated that the mentioned ineffectiveness involves the mechanisms following cell/toxin interactions, or simply because patients are slow to seek medical help. The early events of unregulated inflammatory response and dermonecrosis start to develop in the first 4–8 h after the accidents so that a delay in initiating the use of serum therapy no longer results in the neutralization of the venom toxins [1,2,42,43]. The ineffectiveness of the anti-*Loxosceles* serum therapy in cutaneous loxoscelism could be understood based on the cell biology mechanisms associated to the envenoming. One of the possible explanations lies in the inability of the anti-toxins antibodies to neutralize the toxins after activation or induction of molecular changes in the main target-cells (endothelial cells of the blood vessels, fibroblasts, platelets, and erythrocytes) [20]. Another possibility is that the dermonecrotic toxins (PLDs), which cause almost all of the effects triggered by crude venoms, represent between 15 and 20% of toxin-encoding transcripts in the venom-producing glands. This could generate a low proportional concentration of PLD-neutralizing antibodies in the serum when compared to the high proportional concentration of neutralizing-antibodies for other toxins, resulting in an ineffective serum. If the mentioned hypothesis is true, the production of polyclonal sera with crude venoms enriched with recombinant PLDs or sera obtained using only PLDs as antigens could have a higher neutralizing antibody titer and greater efficiency in serum therapy.

In this work, our main objective was to generate molecular constructions for recombinant expression of PLDs found in the venoms of *L. laeta* and *L. gaucho,* two species of brown spiders with medical importance and endemic to many South American countries. The mutant isoforms lacking biological activity can be used as antigens in high concentrations to obtain an improved serum or vaccine or in other biotechnological applications. Furthermore, these mutant toxins are valuable biotools that can be used to understand PLDs structure-function relationship. The standardization of the cloning, heterologous recombinant expression and purification processes of the recombinant PLDs described herein is a major step since it shows that these toxins can be easily obtained in large scale. PLDs present in the venoms of *L. laeta*, *L. intermedia,* and *L. arizonica* had their structures unveiled at tridimensional level by crystallography approaches [9,15,31,32]. These structural data enabled a better understanding of the mechanisms involved in the cleavage of lipid substrates as well as an identification of the amino acid residues located in functionally important intramolecular sites/regions. Based on the data about the amino acid residues involved in PLDs functionality, we designed constructions encoding wild type and mutant PLDs for both *L. laeta* and *L. gaucho*. The selected mutations comprise amino acid residues located in the catalytic site (H12A-H47A), in the regulatory exosite involved in substrate binding (Y228A and Y228A-Y229A-W230A) and in the magnesium ion coordinating site (E32A-D34A) [15,20,24,31]. With the exception of E32A-D34A mutation for *L. laeta*, all of the designed forms (wild type and mutants) were expressed and purified as soluble proteins (Figure 2). The production of LlRecDT1 E32A-D34A as a soluble protein was unsuccessful despite the numerous attempts (data not shown). The electrophoretic pattern of all wild type and expressed mutant PLDs showed no changes in the migratory profile (such as drag down, aggregations, or fragmentations) nor significant variations in the expected molecular masses (Figure 2), which suggests that mutations did not alter the physicochemical behavior of these molecules. In addition, circular dichroism data pointed out no significant changes in the overall structure of the recombinant PLDs (Figure 3). These data are in line with previous studies that generated mutated isoforms of *Loxosceles intermedia* PLDs [24,44] sharing the same secondary structures profile or spatial conformation/folding of the wild type forms. The comparative molecular modeling and the analysis of the surface electrostatic charges indicated that there are no significant differences between wild type and mutated isoforms for both PLDs of *L. laeta* and *L. gaucho*, except for a slight reduction in the surface negative charges of *L. gaucho* E32A-E34A (Figure 4C). These results strengthen the experimental data acquired in circular dichroism technique, pointing out that the spatial molecular structures did not undergo significant changes after mutations.

In the immunoassays, the highest recognition pattern of the recombinant toxins by the sera raised against the native toxins of their counterpart venom, although may result from different affinities of the respective antibodies, suggests the existence of different epitopes between toxins from *L. gaucho* and *L. laeta* (Figure 5B–D). These data support the idea that protocols for the production of broad-spectrum neutralizing antivenom sera or protective vaccines using recombinant PLDs as antigens must contain recombinant toxins from both venoms, which can assure high neutralization or protection effectiveness following envenomation, especially in regions where both spider species are endemic such as South America.

One of the major challenges for the production of neutralizing antivenom sera and protective vaccines is the choice of antigens. Considering the production of neutralizing hyperimmune sera in animals, it is important that these antigens do not cause great toxic effects in the inoculated animals. The same mindset should be taken into account for antigens used for vaccines. For this reason, the constructions used in this work were designed to generate antigens that meet all of the desirable requirements, i.e., immunogenicity and low toxicity. As observed by spectrofluorimetry and HTPLC, the mutations H12A-H47A, E32A-D34A, and Y228A-Y229A-W230 were extremely efficient in inactivating the PLDs, resulting in only residual sphingomyelin cleavage. On the other hand, the mutation of a single tyrosine at position 228, for both *L. laeta* and *L. gaucho* PLDs, was not sufficient to inhibit their biochemical properties, indicating that enzymes containing this mutation are not useful for serum-development. Some previous data in the literature have shown that the biochemical activities of PLDs in spider venoms, such as sphingomyelin or lysophosphatidylcholine cleavage, and biological activities, such as dermonecrosis and hemolysis, are deeply dependent on the catalytic activities of these enzymes. Inactive or artificial mutant isoforms that do not degrade sphingomyelin also do not induce dermonecrosis or hemolysis [23,24,33,44]. When PLDs act upon sphingomyelin or lysophosphatidylcholine present in the membranes of target cells the lipid mediators formed after enzymatic cleavage constitute biologically active molecules and activate the molecular machinery responsible for the signs and symptoms observed after envenomation [20,45,46]. In this sense, after identifying the inhibition of sphingomyelin-cleavage activity in the mutant isoforms H12A-H47A, E32A-D34A, and Y228A-Y229A-W230, we performed biological analyses regarding well-described activities of these enzymes. The mutated PLDs H12A-H47A, E32A-D34A, and Y228A-Y229A-W230 resulted in loss of hemolytic activity, unchanged capillary permeability, strongly reduced signs of dermonecrosis, and abrogated inflammatory response when compared to the wild type isoforms, strongly supporting a correlation between catalytic activity, dependence on ion metal coordination and substrate binding ability with the noxious effects induced by these enzymes/toxins. These data strongly suggest that the conformational epitopes of mutated isoforms that can act as antigenic determinants have not undergone significant changes, when compared to antigenic determinants of wild type isoforms, enabling any of these mutated variants to be used as antigens in the production of neutralizing hyperimmune sera for treatment of injured victims or of protective vaccines.

## 5. Conclusions

Data shown here describe the production of soluble recombinant PLDs isoforms from *Loxosceles* spider venoms that can be produced in large quantities by using reasonably cost-effective techniques. Additionally, these recombinant mutant PLDs do not exhibit toxic activities, and maintain some of their immunogenic properties, which is desirable for immunotherapeutic purposes. As discussed throughout the text, these mutated recombinant toxins can be used in their pure forms and at high concentrations to generate high-titer hyperimmune sera, which hopefully will recognize wild type native PLDs present in the crude venoms. As a consequence of this recognition, the hyperimmune sera may neutralize the harmful activities caused by the envenomation. These mutant PLDs lacking biological activities are also potential biotools that could be used as antigens for the production of protective vaccines in regions where *Loxosceles* spiders are endemic and therefore cause a great deal of accidents.

## Figures and Tables

**Table 1:** Mutagenic oligonucleotides designed for the generation of site-directed mutations. The codons encoding residues of alanine are underlined.

Species	Mutation	Oligonucleotide Sequence (5′-3′ Sense)
*Loxosceles laeta*	H12A	GTCCAATTTGGAACCTCGCT**GCC**ATGGTGAACGCTGTTGCACA
	H47A	GCCTACTTACACTTAC**GCC**GGAACGCCTTGCGACT
	Y228A	GGGATTCGGCAAATGGATTTATCAATAAAATT**GCC**TACTGGTCTGTAGACAA
	Y228A-Y229A-W230A	CGGCAAATGGATTTATCAATAAAATT**GCCGCCGCC**TCTGTAGACAAAGTATCAACAACGAAGGC
	E32A-D34A	GGATCTTGGTGCAAACGCATTA**GCC**GCG**GCC**GTTACTTTTAAGGGATCAGTGCC
*Loxosceles gaucho*	H12A	CCTATATGGGTTATGGGT**GCC**ATGGTTAACTCCCTCGCT
	H47A	AGCTAATCCTGAATACACATAC**GCC**GGAATTCCCTGCGATTGTGGA
	Y228A	GCGGGATCATTAACAAAGTG**GCC**TATTGGACAGTGGACAAACG
	Y228A-Y229A-W230A	GAAGCGGGATCATTAACAAAGTG**GCCGCCGCC**ACAGTGGACAAACGCCAATCGACAAG
	E32A-D34A	GCCTTGGATCGAATTCAATC**GCC**ACA**GCC**GTGTCATTCGATAAGCAAGC

## Data Availability

Data are contained within the article.
